# Supplementary material for: Zinc Starvation Induces Cell Wall Remodeling and Activates the Antioxidant Defense System in Fonsecaea pedrosoi
Source: J Fungi (Basel). 2024 Jan 31;10(2):118. doi: 10.3390/jof10020118 (PMC10890210; doi:10.3390/jof10020118)
Supplement: Supplementary file 1 [file jof-10-00118-s001.zip › jof-2821678-supplementary.pdf]

Supplementary Materials

Table S1. Oligonucleotides used in qRT-PCR experiments.

ID	Description	Forward	Reverse
Z517_06694	*zrfB*	TGTTAGCTCCTGCCAACGAG	CGCCGTGGTCATGAGCATG
Z517_02544	*zrfA*	CCGGAGTCATTGTCGCCAC	AAGAGGAAGATCACGACCAC
Z517_04287	*zrfC*	AATCATTCCTCCGTCGAGTAC	CCTGTCACAGCGGTAGTTTG
Z517_04288	*pra1*	GGAGAGAATGCCACCGACG	TGGCGGGCAAATGGTAAAGG
Z517_02780	*zrcC*	GCTCGTCCTCATTGTCGGC	CGACCCTTTGTGTCTCCCC
Z517_05482	*β-tub*	CTCCTCTTACTAGCCGCAAC	GGCCCAGTTGTTTCCAGCA

Table S2. Up-regulated proteins in *F. pedrosoi* cells subjected to zinc deprivation for 48 h.

Gene ID	Description	EC number	Score	Fold
**ENERGY**				
**Glycolysis and gluconeogenesis**				
Z517_07053	2-phosphoglycerate dehydratase	4.2.1.11	9459,72	1,323129814
Z517_04938	Glyceraldehyde-3-phosphate dehydrogenase	1.2.1.12	19656,62	2,054433269
**Pentose-phosphate pathway**				
Z517_02720	Glucose-6-phosphate dehydrogenase	1.1.1.49	1440,53	1,433329435
Z517_11222	Phosphoketolase	4.1.2.9	4739	1,786038401
Z517_06162	Ribokinase	2.7.1.15	559,34	#
Z517_11056	Transaldolase	2.2.1.2	9870,97	1,377127754
Z517_01503	Transketolase	2.2.1.1	7583,95	1,323129814
**Tricarboxylic-acid pathway**				
Z517_02370	Acetyltransferase component of pyruvate dehydrogenase complex	2.3.1.12	2900,23	1,462284582
Z517_06504	D-lactate dehydrogenase	1.1.2.4	648,22	#
Z517_08291	Pyruvate dehydrogenase E1 component subunit alpha	1.2.4.1	1612,7	1,390968147
Z517_04045	Pyruvate dehydrogenase E1 component subunit beta	1.2.4.1	1375,4	1,349858824
Z517_00536	Succinate-CoA ligase subunit beta	6.2.1.5	845,35	#
**Electron Transport and Membrane-associated Energy Conservation**				
Z517_05932	ATP synthase subunit beta	7.1.2.2	14892,2	1,41906754
Z517_08312	Cytochrome b5	1.1.2.3	1798,55	#
**METABOLISM**				
**Amino acid metabolism**				
Z517_04289	Aromatic-L-amino-acid decarboxylase	4.1.1.28	788,89	#
Z517_04002	Fumarylacetoacetate hydrolase	3.7.1.2	1025,08	1,390968147
A0A0D2DZ78[§]	Methylthioribose-1-phosphate isomerase	5.3.1.23	833,19	#
Z517_01780	Phosphoserine phosphatase	3.1.3.3	809,64	1,284025417
Z517_07002	O-acetylhomoserine (Thiol)-lyase	2.5.1.47	5770,22	1,568312167
Z517_07495	Methylmalonate-semialdehyde dehydrogenase	1.2.1.27	2343,05	1,41906754

**Nucleotide metabolism**				
Z517_07143	ATP phosphoribosyltransferase	2.4.2.17	604,63	#
Z517_11584	Dihydroorotase	3.5.2.3	1011,31	#
Z517_09914	Orotidine 5'-phosphate decarboxylase	4.1.1.23	651,05	#
Z517_05345	Uracil phosphoribosyltransferase	2.4.2.9	1365,06	2,386910865
**C-compound and carbohydrate metabolism**				
Z517_11479	β-1,3-glucanosyltransferase	2.4.1.-	1013,71	#
Z517_07514	Acetyl-coenzyme A synthetase	6.2.1.1	481,6	#
Z517_05925	α-1,4-glucan branching enzyme	2.4.1.18	746,08	#
Z517_08594	Glycogen phosphorylase	2.4.1.1	1357,55	1,491824707
Z517_06539	D-xylose reductase	1.1.1.307	1054,66	1,447734622
Z517_10903	Formate dehydrogenase	1.17.1.9	1277,24	#
Z517_12498	Formyltetrahydrofolate deformylase	3.5.1.10	3896,69	1,309964465
Z517_06992	Glycogen [starch] synthase	2.4.1.11	927,16	#
Z517_10828	Mannosyl-oligosaccharide glucosidase	3.2.1.106	550,2	#
Z517_10845	NADP-dependent mannitol dehydrogenase	1.1.1.289	7539,08	2,1382762
Z517_00212	Phosphoglycolate phosphatase	3.1.3.18	6250,15	1,349858824
Z517_01565	Aldehyde dehydrogenase	1.2.1.3	2702,82	1,733253039
**Lipid, fatty acid and isoprenoid metabolism**				
Z517_00830	3-hydroxyacyl-ACP-dehydratase	4.2.1.59	1120,73	1,934792385
Z517_07397	3-oxoacyl-ACP-reductase	1.1.1.100	2364,52	#
Z517_03050	3-oxoacyl-ACP-reductase	1.1.1.100	660,18	#
Z517_00829	Fatty acid synthase subunit alpha	2.3.1.86	1179,43	#
Z517_05143	7-alpha-hydroxysteroid dehydrogenase	1.1.1.159	946,34	1,568312167
Z517_04482	Acyl-CoA dehydrogenase	1.3.8.7	699,69	#
Z517_08444	Acyl-CoA dehydrogenase	1.3.8.7	1006,03	#
Z517_01009	Diphosphomevalonate decarboxylase	4.1.1.33	624,23	#
Z517_06161	Enoyl-CoA hydratase	4.2.1.17	1035,9	#
Z517_04197	Enoyl-CoA hydratase		1360,54	1,915540783
Z517_10298	Acyl-protein thioesterase	3.1.2.22	839,61	#
**Secondary metabolism**				

Z517_07016	Dienelactone hydrolase	3.1.1.-	4965,09	#
**CELL CYCLE AND DNA MAINTENANCE**				
**Cell cycle**				
Z517_06757	Sexual development protein		621,92	#
**DNA maintenance**				
Z517_03993	DNA damage-inducible protein 1		595,36	#
Z517_11645	Kelch repeat protein		4281,64	#
Z517_09628	Methyltransferase		655,9	#
Z517_00871	N-acetyltransferase domain-containing protein		862,41	#
Z517_04847	Tetratricopeptide repeat protein 1	3.4.25.1	558,18	#
**TRANSCRIPTION**				
**Transcription control**				
Z517_10876	C6 transcription factor		1821,04	#
Z517_07226	NmrA domain-containing protein		807,15	#
**PROTEIN SYNTHESIS**				
**Translation control**				
Z517_11361	Endoribonuclease L-PSP	3.5.99.10	1583,63	#
**Ribosome biogenesis**				
Z517_04306	40S ribosomal protein S24		1697,26	#
Z517_03437	54S ribosomal protein L28, mitochondrial		452,13	#
**PROTEIN FATE and DEGRADATION**				
**Protein modification**				
Z517_07815	Protein disulfide-isomerase	5.3.4.1	3973,49	1,349858824
**Protein degradation**				
Z517_06176	Aminopeptidase	3.4.11	521,59	1,221402762
Z517_01254	Dipeptidyl peptidase 3	3.4.14.4	1599,45	1,363425117
Z517_10857	E3 ubiquitin ligase complex SCF subunit		973,86	#
Z517_04931	Insulysin	3.4.24.56	2801,73	14,15404

Z517_04037	Octapeptidyl aminopeptidase	3.4.24.59	919,1	1,336427477
Z517_04034	Proteasome subunit alpha type	3.4.25.1	934,27	#
Z517_10340	Proteasome subunit alpha type	3.4.25.1	1259,63	1,271249144
Z517_02221	Proteasome subunit beta type-6	3.4.25.1	999,31	#
**CELLULAR TRANSPORT, TRANSPORT FACILITATION AND TRANSPORT ROUTES**				
Z517_10008	Ran GTPase-activating protein 1		727,54	#
Z517_03308	V-type proton ATPase		493,62	#
**CELLULAR COMMUNICATION/SIGNAL TRANSDUCTION MECHANISM**				
Z517_09053	Histidine phosphotransferase		2442,07	#
**CELL RESCUE, DEFENSE AND VIRULENCE**				
**Stress response**				
Z517_10553	Hsp12		11157,11	1,349858824
Z517_12187	Hsp88		2785,96	1,296930074
**Detoxification**				
Z517_06195	Catalase	1.11.1.6	7858,18	#
Z517_04832	Glutathione reductase	1.8.1.7	668,97	1,296930074
Z517_10658	Glutathione synthetase	6.3.2.3	630,87	1,390968147
Z517_05334	Glutathione-S-transferase	2.5.1.18	865,53	#
Z517_09629	Superoxide dismutase [Fe-Mn]	1.15.1.1	7185,27	#
Z517_01853	Hydroxyacylglutathione hydrolase	3.1.2.6	791,34	#
**UNCLASSIFIED**				
Z517_00761	Uncharacterized protein		730,3	#
Z517_02139	Uncharacterized protein		8542,49	1,521961536
Z517_02301	Uncharacterized protein		3819,54	#
Z517_02398	Uncharacterized protein		2461,59	1,682027618
Z517_02558	Uncharacterized protein		476,65	#
Z517_02972	Uncharacterized protein		820,52	#
Z517_03128	Uncharacterized protein		781,82	#
Z517_03251	Uncharacterized protein		821,34	#
Z517_03763	Uncharacterized protein		1215,97	#

Z517_04379	Uncharacterized protein	3353,39	1,568312167
Z517_04428	Uncharacterized protein	1618,19	1,50681778
Z517_04542	Uncharacterized protein	618,46	#
Z517_04787	Uncharacterized protein	518,82	#
Z517_04901	Uncharacterized protein	1317,6	#
Z517_05050	Uncharacterized protein	713,45	#
Z517_05205	Uncharacterized protein	1991,14	#
Z517_05526	Uncharacterized protein	2952,59	#
Z517_05809	Uncharacterized protein	550,51	#
Z517_06207	Uncharacterized protein	759,6	#
Z517_06322	Uncharacterized protein	630,69	#
Z517_06538	Uncharacterized protein	1158,26	#
Z517_07726	Uncharacterized protein	12800,81	3,320117081
Z517_08120	Uncharacterized protein	785,73	#
Z517_09135	Uncharacterized protein	590,16	#
Z517_10304	Uncharacterized protein	711,81	#
Z517_10328	Uncharacterized protein	1353,05	#
Z517_10430	Uncharacterized protein	4667,35	1,447734622
Z517_10960	Uncharacterized protein	999,01	1,377127754
Z517_11503	Uncharacterized protein	12485,27	1,363425117
Z517_12095	Uncharacterized protein	685,84	#
Z517_12150	Uncharacterized protein	667,76	#
Z517_12367	Uncharacterized protein	1249,67	#
Z517_03907	Uncharacterized protein	3105,86	1,632316236
Z517_01609	Uncharacterized protein	719,94	1,233678052
Z517_01042	Uncharacterized protein	727,81	#
Z517_02450	Uncharacterized protein	2462,57	#
Z517_12018	Uncharacterized protein	959,54	1,284025417
Z517_12249	Uncharacterized protein	2797,6	1,323129814
Z517_12281	Uncharacterized protein	640,67	#
Z517_09962	Uncharacterized protein	1879,95	1,41906754

§ Uniprot input codes.

# Proteins detected only in DTPA condition at the time point of 48 h.

Table S3. Down-regulated proteins in *F. pedrosoi* cells subjected to zinc deprivation for 48 h.

Gene ID	Description	EC number	Score	Fold
**ENERGY**				
**Glycolysis and gluconeogenesis**				
Z517_03031	Fructose-bisphosphate aldolase	2.1.1.127	5746,52	0,733446954
Z517_02120	Glucose-6-phosphate epimerase	5.1.3.15	3046,02	0,810584251
Z517_11930	Phosphoglycerate mutase	5.4.2.11	3705,33	0,778800783
**Pentose-phosphate pathway**				
Z517_00790	6-phosphogluconate dehydrogenase	1.1.1.44	759,99	0,491644208
Z517_04020	Ribose 5-phosphate isomerase	5.3.1.6	651,29	#
**Tricarboxylic-acid pathway**				
Z517_11857	Aconitase	4.2.1.3	1202,59	0,748263574
Z517_01790	ATP citrate synthase	2.3.3.8	970,68	0,810584251
Z517_11581	Dihydrolipoyl dehydrogenase	1.8.1.4	892,43	0,063927861
Z517_10399	Fumarate hydratase	4.2.1.2	1741,92	0,810584251
Z517_00815	Fumarate reductase	1.3.1.6	582,73	#
Z517_02225	Isocitrate dehydrogenase, subunit 1	1.1.1.41	1401,79	0,778800783
Z517_08788	Isocitrate dehydrogenase, subunit 2	1.1.1.41	1414,18	0,537944435
Z517_11140	Isocitrate dehydrogenase	1.1.1.42	507,97	#
Z517_07774	Malate dehydrogenase	1.1.1.37	6015,34	0,726149042
**Electron transport and membrane-associated energy conservation**				
Z517_06346	Cytochrome c oxidase subunit 6, mitochondrial	7.1.1.9	1656,6	0,690734327
Z517_03156	Flavocytochrome c	1.3.4.1	674,2	#
Z517_11785	NADH-cytochrome b5 reductase	1.6.2.2	529,7	#
Z517_12315	Electron transfer flavoprotein subunit alpha	1.5.5.1	930,81	#
Z517_10858	Electron transfer flavoprotein subunit beta	1.5.5.1	2704,7	0,559898376
**Fermentation**				
Z517_05281	Alcohol dehydrogenase	1.1.1.1	931,1	#
Z517_11468	Pyruvate decarboxylase	4.1.1.1	540,3	#

METABOLISM

Amino acid metabolism

A0A0D2GZG5§	1,2-dihydroxy-3-keto-5-methylthiopentene dioxygenase	1.13.11.53	1250,09	0,637628159
Z517_07947	2-isopropylmalate synthase	2.3.3.13	615,6	#
Z517_04233	3-isopropylmalate dehydratase	4.2.1.33	961,4	#
Z517_00179	4-aminobutyrate transaminase	2.6.1.19	989,79	0,440431658
A0A0D2GXZ8§	Alanine--tRNA ligase	6.1.1.7	898,9	0,618783398
Z517_05830	Amine oxidase	1.4.3.4	718,52	#
Z517_07507	Aminotransferase class I/classII	2.3.1.29	480,37	#
Z517_01180	Anthranilate phosphoribosyltransferase	2.4.2.18	522,65	#
Z517_08456	Arginosuccinase	4.3.2.1	1904,98	0,670320042
Z517_05615	Asparagine synthase	6.3.5.4	718,93	#
Z517_03823	Branched-chain amino acid aminotransferase	2.6.1.42	488,37	#
Z517_01314	Branched-chain-amino-acid aminotransferase	2.6.1.42	842,04	#
Z517_05619	Chorismate synthase	4.2.3.5	541,98	#
Z517_11463	Dihydroxy-acid dehydratase	4.2.1.9	644,15	#
Z517_11386	Glutamate decarboxylase	4.1.1.15	1100,29	#
Z517_06406	Glutamate synthase (NADH)	1.4.1.14	1277,97	#
Z517_09829	Glutamine synthetase	6.3.1.2	1679,93	0,543350861
Z517_08362	Imidazole glycerol phosphate synthase hisHF	4.3.2.10	778,53	0,548811623
Z517_11990	Ketol-acid reductoisomerase_ mitochondrial	1.1.1.86	5713,8	0,657046828
A0A0D2GGE0§	Methylthioribulose-1-phosphate dehydratase	4.2.1.109	933,44	#
Z517_07074	Ornithine aminotransferase	2.6.1.13	480,18	#
Z517_04109	Phospho-2-dehydro-3-deoxyheptonate aldolase	2.5.1.54	431,99	0,394553708
Z517_03204	Lysine-2-oxoglutarate reductase	1.5.1.7	552,14	#
Z517_11246	Serine hydroxymethyltransferase	2.1.2.1	3561,32	0,810584251
Z517_10247	Serine hydroxymethyltransferase	2.1.2.1	478,4	#
Z517_09806	Spermidine synthase	2.5.1.16	1598,49	#
Z517_09767	Trihydroxynaphthalene reductase	2.7.1.39	6983,4	0,637628159

Nitrogen and sulfur metabolism

Z517_04082	2-nitropropane dioxygenase	1.6.5.9	691,01	0,472366553
Z517_05518	Adenylylsulfate kinase	2.7.1.25	3273,18	#
Z517_04891	Sulfite reductase	1.8.1.2	778,68	#
Z517_08191	Sulfite reductase	1.8.1.2	469,01	0,527292432

Z517_10130	Methionine synthase	2.1.1.14	13198,19	0,794533599
Z517_11935	Cystathionine beta-synthase	4.2.1.22	566,14	#
Z517_09326	S-adenosylmethionine synthase	2.5.1.6	2269,12	0,810584251
Z517_01491	S-adenosylmethionine synthase	2.5.1.6	5653,63	0,778800783
**Nucleotide metabolism**				
Z517_07655	Adenine phosphoribosyltransferase	2.4.2.7	1203,09	#
Z517_02903	Carbamoyl-phosphate synthase arginine-specific large chain	6.3.5.5	654,83	0,697676316
Z517_05552	Dihydroorotate dehydrogenase	1.3.98.1	621,97	#
Z517_00048	Inosine-5'-monophosphate dehydrogenase	1.1.1.205	2889,59	#
Z517_10346	Phosphoribosylamine-glycine ligase	6.3.4.13	796,29	#
**C-compound and carbohydrate metabolism**				
Z517_12153	Chlorophyll synthesis pathway protein BchC	1.1.1.287	3278,14	#
Z517_01036	FGGY-family pentulose kinase	2.7.1.47	587,22	#
Z517_10609	Haloacid dehalogenase, type II	3.8.1.2	857,7	#
Z517_03785	Isopropylmalate dehydrogenase	1.1.1.85	629,01	#
Z517_12474	S-(hydroxymethyl)glutathione dehydrogenase	1.1.1.284	524,71	#
Z517_02033	Succinate-semialdehyde dehydrogenase	1.2.1.16	484,05	#
3Z517_02502	Malic enzyme	1.1.1.38	1354,73	0,718923724
Z517_08203	Methyltransferase	2.1.1.-	796,67	#
Z517_03465	Aldehyde dehydrogenase	1.2.1.3	2050,09	0,778800783
**Lipid, fatty acid and isoprenoid metabolism**				
Z517_08709	Choline/carnitine acyltransferase	2.3.1.21	454,43	#
Z517_11422	Enoyl reductase (ER)	1.6.5.5	2300,2	0,143703942
Z517_03788	Glycerol-3-phosphate dehydrogenase	1.1.1.8	621,31	#
Z517_07094	Inositol-3-phosphate synthase	5.5.1.4	1941,51	0,718923724
Z517_04924	Isopentenyl-diphosphate Delta-isomerase	5.3.3.2	595,44	#
Z517_11087	Phytanoyl-CoA dioxygenase		546,63	#
**Metabolism of vitamins, cofactors and prosthetic groups**				
Z517_11513	Formyltetrahydrofolate synthetase	6.3.4.3	862,33	0,644036423
Z517_01805	Hydroxymethylbilane synthase	2.5.1.61	549,96	#

Z517_08984	Pyridoxal 5'-phosphate synthase		722,28	0,65050909
Z517_10905	Pyridoxal 5'-phosphate synthase		1472,32	0,683861412
Z517_01999	Pyridoxal phosphate homeostasis protein		1397,99	#
Z517_06157	Thiamine thiazole synthase	2.4.2.60	1139,52	0,49658531
**Secondary metabolism**				
Z517_10541	Alpha-ketoglutarate-dependent 2,4-dichlorophenoxyacetate dioxygenase	1.14.11.-	542,68	#
Z517_04492	Aminotransferase class V domain-containing protein	4.4.1.16	592,26	#
Z517_06354	CipC-like antibiotic response protein		2604,56	#
Z517_09985	Scytalone dehydratase	4.2.1.94	4602,82	0,740818212
**CELL CYCLE AND DNA MAINTENANCE**				
**Cytoskeleton**				
Z517_00583	Profilin		1729,59	0,677056884
Z517_03821	Tropomyosin		1709,46	0,440431658
Z517_02603	Tubulin alpha chain		530,93	#
**DNA maintenance**				
Z517_00810	5'-deoxynucleotidase	3.1.3.89	527,33	#
Z517_09643	DNA damage-responsive protein 48		6379,83	0,367879441
Z517_06712	DNA-binding protein		892,4	#
Z517_09820	GrpE protein homolog_ mitochondrial		496,55	0,292292572
Z517_11699	Histone H2B		518,7	#
Z517_03214	Histone H4		1237,13	0,683861412
Z517_08352	Histone H4		1237,13	0,644036423
Z517_11102	HIT domain-containing protein		1141,63	#
Z517_07364	Nuclear segregation protein		1824,94	#
Z517_10216	Nucleosome assembly protein		5371,21	0,683861412
Z517_05416	Obg-like ATPase 1		979,89	#
**TRANSCRIPTION**				
**RNA synthesis and processing**				
Z517_02606	DNA-directed RNA polymerase	2.7.7.6	674,27	#
Z517_08834	Hyaluronan/mRNA-binding		1576,74	0,537944435

Z517_01501	Polyadenylate-binding protein	-	768,97	#
Z517_04829	Ribonucloprotein	-	1637,98	0,771051593
Z517_10455	Ribonucloprotein		8304,19	0,58274824
Z517_04004	RNA helicase	3.6.4.-	475,66	#
Z517_03035	RNA polymerase II degradation factor 1		554,06	#
Z517_02759	Telomere length regulation protein TEL2		605,53	#
**PROTEIN SYNTHESIS**				
**Translation factors**				
Z517_07932	Elongation factor 1-alpha	-	576,35	0,0278757
Z517_05553	Elongation factor 1-beta	-	4120,52	0,677056884
Z517_10365	Elongation factor 1-gamma	-	3703,73	0,511708569
Z517_08555	Elongation factor 2	-	926,58	0,63128364
Z517_08550	Elongation factor 3	-	733,8	#
A0A0D2H139[§]	Eukaryotic translation initiation factor 3 subunit B		718,81	#
Z517_02400	Eukaryotic translation initiation factor 5A		582,12	0,244143291
**Ribosome biogenesis and aminoacyl-tRNA-synthetases**				
A0A0D2GSI6[§]	40S ribosomal protein S0	-	5671,44	0,778800783
A0A0D2GST6[§]	40S ribosomal protein S1	-	4778,84	0,786627865
Z517_02627	40S ribosomal protein S13	-	2679,91	0,763379486
Z517_00568	40S ribosomal protein S14	-	11862,5	0,718923724
Z517_00570	40S ribosomal protein S16	-	1486,42	0,778800783
Z517_00734	40S ribosomal protein S17	-	3134,61	0,818730751
Z517_11882	40S ribosomal protein S19	-	2439,73	0,755783741
Z517_04579	40S ribosomal protein S22	-	2355,12	#
Z517_10683	40S ribosomal protein S23	-	1994,19	0,794533599
Z517_04578	40S ribosomal protein S25	-	6768,57	0,677056884
Z517_07089	40S ribosomal protein S28	-	7193,68	0,818730751
Z517_07142	40S ribosomal protein S28	-	1184,55	0,771051593
Z517_01712	40S ribosomal protein S29	-	7920,16	0,818730751
Z517_08921	40S ribosomal protein S3	-	4412,75	0,71177032
Z517_00799	40S ribosomal protein S5	-	1800,31	0,810584251
Z517_08727	40S ribosomal protein S6	-	2324,72	0,565525443
Z517_07561	40S ribosomal protein S7	-	1516,93	0,786627865

Z517_05597	60S acidic ribosomal protein P1	-	2474,29	0,506616989
Z517_02840	60S acidic ribosomal protein P2	-	14653,56	0,771051593
Z517_11818	60S ribosomal protein L12	-	4367,61	0,826959136
Z517_04802	60S ribosomal protein L23-A	-	4456,3	#
Z517_10751	60S ribosomal protein L24	-	1784,04	0,778800783
Z517_02719	60S ribosomal protein L33-A	-	5258,5	0,704688094
Z517_00991	60S ribosomal protein L35	-	1578,45	#
Z517_00063	60S ribosomal protein L43-B	-	1303,02	0,771051593
Z517_09910	60S ribosomal protein L44	-	2528,81	#
Z517_12164	60S ribosomal protein L5	-	5570,86	0,748263574
Z517_04516	60S ribosomal protein L6	-	3621,53	0,794533599
Z517_05541	60S ribosomal protein L9-B	-	1857,56	0,637628159
Z517_12464	60SRibosomal protein L22e	-	6118,22	0,637628159
Z517_10495	Ribosomal L28e/Mak16	-	4549,19	0,763379486
Z517_00980	Ribosomal protein	-	2161,07	0,763379486
Z517_00419	Ribosome maturation protein SDO1/SBDS*	-	2761,13	0,571209062
Z517_06716	Asparaginyl-tRNA synthetase	6.1.1.22	775,55	0,763379486
Z517_05721	Aspartyl-tRNA synthetase	6.1.1.12	809,4	0,410655759
Z517_10378	Cysteinyl-tRNA synthetase	6.1.1.16	657,22	#
Z517_00506	Glutamyl-tRNA synthetase	6.1.1.17	459,14	0,733446954
Z517_10186	Glycine-tRNA ligase	6.1.1.14	707,91	0,199887611
Z517_09088	Leucyl-tRNA synthetase	6.1.1.4	619,29	0,394553708
Z517_01593	Prolyl-tRNA synthetase	6.1.1.15	544,87	#
Z517_10236	Seryl-tRNA synthetase	6.1.1.11	548,95	#
Z517_11519	Threonyl-tRNA synthetase	6.1.1.3	475,01	#
Z517_11158	Tryptophanyl-tRNA synthetase	6.1.1.2	1538,82	#
Z517_07860	Tyrosine--tRNA ligase	6.1.1.1	821,2	#
Z517_03071	Valyl-tRNA synthetase	6.1.1.9	585,24	#
**PROTEIN FATE and DEGRADATION**				
**Protein modification**				
Z517_07382	Protein arginine methyltransferase	2.1.1.-	834,93	0,379083027
Z517_11352	Mitochondrial-processing peptidase subunit beta		486,23	#
Z517_00023	Peptidyl-prolyl cis-trans isomerase	5.2.1.8	2819,27	0,594520559
Z517_01406	Protein disulfide-isomerase domain	5.3.4.1	714,25	#

Z517_11074	Subtilisin-like protease		665,12	0,472366553
**Protein degradation**				
Z517_03382	26S protease regulatory subunit 6A		526,39	0,543350861
Z517_10325	Aspartyl aminopeptidase	3.4.11.21	602,36	#
Z517_11678	Peptidase A1		967,69	#
Z517_02563	Polyubiquitin		3541,21	0,683861412
Z517_07149	Proteasome endopeptidase complex	3.4.25.1	566,06	#
Z517_09646	Proteasome	3.4.25.1	668,22	#
Z517_08401	Ubiquitin		3541,21	0,683861412
Z517_12078	Ubiquitin-40S ribosomal protein S27a		3541,21	0,690734327
**CELL RESCUE, DEFENSE AND VIRULENCE**				
**Stress response**				
Z517_05127	10 kDa heat shock protein, mitochondrial		7466,39	0,740818212
Z517_05928	HSP 60-like protein		3488,98	0,748263574
Z517_06172	HSP 72-like protein		3451,62	0,588604987
Z517_12374	Hsp7		5949,09	0,726149042
Z517_05642	Hsp70 chaperone		1109,76	#
Z517_03121	HSP70-like protein		1681,68	0,49658531
Z517_06860	Stress protein DDR48		7754,78	0,440431658
**Detoxification**				
Z517_10212	Superoxide dismutase [Cu-Zn]	1.15.1.1	2382,99	0,292292572
Z517_01433	Thioredoxin	1.8.1.9	5716,46	0,786627865
**CELLULAR TRANSPORT, TRANSPORT FACILITIES AND TRANSPORT ROUTES**				
Z517_00982	ADP-ribosylation factor		1640,23	#
Z517_12486	Endoplasmic reticulum chaperone BiP	3.6.4.10	1047,51	0,794533599
Z517_00412	GTP-binding nuclear protein		6098,65	0,612626388
Z517_10740	Mitochondrial import inner membrane translocase subunit		788,49	#
Z517_08559	Nascent polypeptide-associated complex subunit alpha		2095,06	0,506616989
**UNCLASSIFIED**				

Z517_00220	Uncharacterized protein	878,91	0,594520559
Z517_00235	Uncharacterized protein	918,27	#
Z517_00315	Uncharacterized protein	975,43	#
Z517_00336	Uncharacterized protein	466,67	#
Z517_00726	Uncharacterized protein	549,12	#
Z517_01038	Uncharacterized protein	2483,28	0,390627836
Z517_01144	Uncharacterized protein	6390,82	0,58274824
Z517_01274	Uncharacterized protein	1783,74	#
Z517_01372	Uncharacterized protein	1002,62	0,663650253
Z517_01422	Uncharacterized protein	1079,68	0,576949804
Z517_01803	Uncharacterized protein	487,94	#
Z517_01901	Uncharacterized protein	880,68	0,65050909
Z517_02883	Uncharacterized protein	637,48	#
Z517_03130	Uncharacterized protein	513,22	#
Z517_03672	Uncharacterized protein	2778,73	0,786627865
Z517_03816	Uncharacterized protein	618,58	#
Z517_03890	Uncharacterized protein	573,84	0,576949804
Z517_03976	Uncharacterized protein	1443,11	#
Z517_04283	Uncharacterized protein	605,84	#
Z517_05221	Uncharacterized protein	2237,38	0,786627865
Z517_05545	Uncharacterized protein	610,84	0,697676316
Z517_05857	Uncharacterized protein	670,95	#
Z517_06999	Uncharacterized protein	704,36	#
Z517_07958	Uncharacterized protein	2813,17	0,778800783
Z517_08054	Uncharacterized protein	751,86	#
Z517_09508	Uncharacterized protein	457,7	#
Z517_09561	Uncharacterized protein	494,48	#
Z517_09825	Uncharacterized protein	1356,94	#
Z517_11207	Uncharacterized protein	928,99	0,748263574
Z517_11609	Uncharacterized protein	894,13	#
Z517_12076	Uncharacterized protein	1250,56	0,625002269
Z517_12501	Uncharacterized protein	512,54	#

[§] Uniprot input codes.

[#] Proteins detected only in control condition at the time point of 48 h.
